# Susceptibility of Fibromatosis Cells in Short-Term Culture to Ifosfamide: A Possible Experimental Treatment in Clinically Aggressive Cases

**DOI:** 10.1080/13577149977686

**Published:** 1999-09

**Authors:** Mark W. Verrill, Helen M. Coley, Ian R. Judson, Cyril Fisher

**Affiliations:** 1 University of Newcastle Department of Medical Oncology Newcastle General Hospital Westgate Road Newcastle upon Tyne NE4 6B E UK; 2 CRC Centre for Cancer Therapeutics Institute of Cancer Research 15 Cotswold Road Belmont, Sutton Surrey SM2 5NG UK; 3 Sarcoma Unit Royal Marsden NHS Trust Fulham Road London SW3 6JJ UK

## Abstract

*Purpose.* Deep fibromatoses are large, often rapidly growing but benign soft tissue tumours. Although surgery is the mainstay of treatment, in unremitting and aggressive cases the use of cytotoxic chemotherapy may produce objective tumour responses. Fresh tumour samples from four patients with fibromatosis were investigated as part of a study of drug resistance in soft tissue tumours.

*Methods.* Following short-term culture of fibromatosis cells *in vitro* , chemosensitivity to 4-hydroperoxy-ifosfamide, the active form of ifosfamide and doxorubicin was tested. Following 72-h continuous exposure to each drug, surviving cell fraction was assessed using the lactate dehydrogenase assay.

*Results.* Mean IC_50_ values for ifosfamide and doxorubicin were 6.2 and 0.35 µmol/l, respectively. In samples of soft tissue sarcoma (STS) from the same study the mean IC_50_ values for ifosfamide and doxorubicin were 14.8 and 1.69 µmol. The difference in mean ifosfamide IC_50_ values for fibromatosis and STS samples was statistically significant.

*Discussion.* We are not aware of any other report suggesting the use of ifosfamide in this condition. These observations suggest that, for patients with inoperable or progressive lesions of fibromatosis causing significant morbidity, it may be valuable to include ifosfamide in experimental treatment regimens.


*Sarcoma* (1999) **3**, 79–84

ORIGINAL ARTICLE

### Susceptibility of fibromatosis cells in short-term culture to ifosfamide: a possible experimental treatment in clinically aggressive cases

MARK W. VERRILL,[1] HELEN M. COLEY,[2] IAN R. JUDSON[2,3] & CYRIL FISHER[3]

[1]*University of Newcastle Department of Medical Oncology, Newcastle General Hospital, Westgate Road, Newcastle upon Tyne, NE4 6BE, UK,* [2]*CRC Centre for Cancer Therapeutics, Institute of Cancer Research, 15 Cotswold Road, Belmont, Sutton, Surrey, SM2 5NG, UK,* [3]*Sarcoma Unit, Royal Marsden NHS Trust, Fulham Road, London, SW3 6JJ, UK*

**Abstract**

*Purpose.* Deep fibromatoses are large, often rapidly growing but benign soft tissue tumours. Although surgery is the mainstay of treatment, in unremitting and aggressive cases the use of cytotoxic chemotherapy may produce objective tumour responses. Fresh tumour samples from four patients with fibromatosis were investigated as part of a study of drug resistance in soft tissue tumours.

*Methods.* Following short-term culture of fibromatosis cells *in vitro*, chemosensitivity to 4-hydroperoxy-ifosfamide, the active form of ifosfamide and doxorubicin was tested. Following 72-h continuous exposure to each drug, surviving cell fraction was assessed using the lactate dehydrogenase assay.

*Results.* Mean $IC_{50}$ values for ifosfamide and doxorubicin were 6.2 and 0.35 μmol/l, respectively. In samples of soft tissue sarcoma (STS) from the same study the mean $IC_{50}$ values for ifosfamide and doxorubicin were 14.8 and 1.69 μmol. The difference in mean ifosfamide $IC_{50}$ values for fibromatosis and STS samples was statistically significant.

*Discussion.* We are not aware of any other report suggesting the use of ifosfamide in this condition. These observations suggest that, for patients with inoperable or progressive lesions of fibromatosis causing significant morbidity, it may be valuable to include ifosfamide in experimental treatment regimens.

**Key words**: *fibromatosis, chemotherapy, ifosfamide, experimental treatment, aggressive disease*

### Introduction

The deep fibromatoses, also known as desmoid tumours, are large, often rapidly growing but benign soft tissue tumours. They are rare with a reported incidence of 0.2–0.5 per 100 000 population per year, most commonly affecting young adults, especially women. Based on their anatomical location, the deep fibromatoses can be divided into three subtypes, extra-abdominal, abdominal and intra-abdominal. Extra-abdominal fibromatosis arises principally from the connective tissue of muscle and the overlying fascia or aponeurosis and chiefly affects the muscles of the shoulder and pelvic girdles and the thigh. Abdominal fibromatosis arises from the musculo-aponeurotic structures of the abdominal wall and tends to occur in women of child bearing age during or after pregnancy, leading to the hypothesis that hormonal factors are important in the aetiology of the condition. Intra-abdominal fibromatosis arises inside the abdominal cavity and includes pelvic and mesenteric fibromatosis and fibromatosis in Gardner's syndrome.[1] Histologically they are all poorly circumscribed infiltrating tumours comprising spindle cells regularly dispersed in collagen, without atypical or hyperchromatic nuclei but with very occasional mitotic figures. The presence of numerous or atypical mitotic figures should arouse suspicion of a malignant lesion.

Following the typical presentation with a soft tissue mass, diagnosis should be confirmed, preferably by needle core biopsy, before treatment is undertaken. Radical surgical excision is the treatment of choice due to the infiltrative nature of the tumour.[2–4] However, because the tumours are non-malignant with potential for prolonged survival, even in the presence of bulky disease, it is desirable to avoid mutilating surgery.[5,6] Local recurrence occurs in approximately 40% of cases rising to 90% after marginal excision.[5,7–10] Radiotherapy has an established role both as an adjunct to surgery and for inoperable cases.[11–14] Tumour regression following radiotherapy may be prolonged.

Spontaneous regression of growth and a 'plateau phase' have been observed in cases of fibromatosis

Correspondence to: I.R. Judson, CRC Centre for Cancer Therapeutics, Institute of Cancer Research, 15 Cotswold Road, Belmont, Sutton, Surrey SM2 5NG, UK. Fax: +44 181 770 7885; E-mail: judson@icr.ac.uk

1357-714X print/1369-1643 online/99/020079-06 © 1999 Taylor & Francis Ltd with residual or inoperable disease after surgery.[5,6] This observation contributes to the reluctance of clinicians to use cytotoxic treatments routinely in the management of an essentially benign disease. Many clinicians feel that the risk of treatment-related side effects, particularly the long-term risk of induction of malignant disease makes this form of treatment unacceptable. However, in unremitting and aggressive cases the use of hormone or cytotoxic chemotherapy can more easily be justified and there are several published reports of the successful use of anti-oestrogens, including toremifene and tamoxifen,[15–18] progesterone,[19] non-steroidal anti-inflammatory prostaglandin inhibiting drugs[16] and various cytotoxic chemotherapy regimens[20–23] to control cases of fibromatosis.

Although many different chemotherapy regimens have been used, there is no clear agreement between investigators on which regimen is best. In this report, we present *in vitro* chemosensitivity data for tumour cells growing in short-term culture derived from samples of excised tumour in four cases of fibromatosis treated by surgery. Tumour cells were exposed to doxorubicin and ifosfamide, the two first line drugs most commonly used in the treatment of adult soft tissue sarcoma (STS) and the results are compared with typical malignant STS samples.

### Methods

### *Patients*

Patients presented here were investigated as part of a research programme within the Institute of Cancer Research and the Royal Marsden Hospital to study mechanisms of drug resistance in patients with soft tissue tumours. This project has been approved by the local ethics committee. Sterile samples of fresh tumour were collected from the operative specimens of patients undergoing primary surgery for soft tissue tumours. These samples were studied in the laboratory and the results were correlated with the clinical outcome of the patients. The four patients in the series found to have a pathological diagnosis of fibromatosis and evaluable for *in vitro* cytotoxicity are described.

### *Processing of tumour material*

Samples of fresh sarcoma tissue were collected under sterile conditions into L15 Leibovitz culture medium (Gibco Life Technologies, Paisley, Scotland) containing the antibiotics gentamicin and amphotericin B. Samples were processed within 48 h of collection as follows: tissue was subjected to mincing using sterile crossed scalpels, followed by digestion at 37°C using collagenase (Sigma Chemicals, Poole) at a working concentration of 500–1000 IU/ml, diluted in L15 medium. The duration of digestion varied according to the rigidity and texture of the sample and was between 30 min and 2 h. Erythrocytes were removed using a density gradient (Histopaque-1077; Sigma Chemicals), and further mechanical disaggregation and cell washing was carried out in order to achieve a suspension with minimal cell clumping and debris.

Fibromatosis cells were cultured in tissue culture flasks and maintained in an environment of 5% carbon dioxide at 37°C, in Ham's F12 medium with Glutamax (Gibco Life Technologies), supplemented with 20% heat-inactivated foetal calf serum (FCS) and the antibiotics gentamicin and amphoteracin B. Cultures were allowed to equilibrate and were subjected to several changes of medium in order to remove further debris over a period of at least 48 h. All cultures grew as attached monolayers. Samples of the fresh tumour cell suspensions initially seeded into tissue culture flasks were used to make cytospin preparations using a Shandon Cytospin 3 (Life Sciences International UK Ltd., Basingstoke). Giemsa stained cytospins were used for verification of tumour cell content which was greater than 80% in all cases.

### *Chemosensitivity testing*

Fresh tumour cells growing as monolayer cultures were trypsinised (trypsin–EDTA, Gibco Life Technologies) to produce a single cell suspension in Ham's F12 medium containing 10% FCS. Cells were dispensed into 96-well tissue culture plates in 200-μl aliquots at a density of $2 \times 10^5$/ml–$1 \times 10^6$/ml.

Cultures were allowed to equilibrate for 24 h in an humidifying gassing incubator. Doxorubicin (Sigma Chemicals), obtained as the hydrochloride salt, was made up in sterile distilled water and stored as frozen aliquots. Ifosfamide (ASTA Medica, Frankfurt, Germany), obtained as the activated 4-hydroperoxy form, was dissolved in sterile distilled water immediately before use. Freshly prepared drug solutions diluted in tissue culture medium containing 10% FCS were dispensed in aliquots to give the desired final concentration, over a dose range of 0.1–2 μM for doxorubicin and 0.5–100 μM for ifosfamide. The cultures were then incubated at 37°C in a humidifying gassing incubator for 72 h, with the cells continuously exposed to the drugs.

Trays of drugs-treated cells were assessed for cell viability using an LDH-release assay kit (Cytotox96, Promega Corporation, Southampton, UK) which we have adapted for use in cytotoxicity testing.[24] Spent tissue culture medium was aspirated and replaced with 200 μl of serum-free phenol red-free Dulbecco's medium (Gibco Life Technologies). Cells were ruptured by freezing to –70°C and then allowing to thaw at room temperature. 100 μl of cell lysate was then removed from each well and dispensed into a duplicate tray. Fifty microlitres of LDH substrate mixture were added to each well and the colour reaction allowed to take place for 30–60 min at room temperature in the dark. Fifty microlitres of 'Stop solution' from the kit were added to each well and colour absorbance at 492 nm (red) was measured using a plate reading spectrophotometer. Absorbance readings of control untreated cell wells were compared to drug-treated wells. Data were calculated as absorbance of test wells relative to control wells (mean of at least three replicates), with $IC_{50}$ denoting the concentration of drug required to cause a 50% loss of cell viability relative to control. These values were calculated from plots of $\log_{10}$ drug concentration ($x$ axis) vs fraction of control absorbance ($y$ axis).

Cytotoxicity testing results for fibromatosis samples were compared with $IC_{50}$ values from 18 evaluable cases of soft tissue sarcoma collected in the same study. The remainder of the STS specimens and the one other fibromatosis specimen collected failed to grow or became contaminated making cytotoxicity testing impossible. None of the patients with STS had previously received chemotherapy. Several of the soft tissue sarcoma specimens had $IC_{50}$ values for ifosfamide and/or doxorubicin above the limit of detection of the cytotoxicity assay. By convention, $IC_{50}$ values for these cases were set at the maximum detectable value, typically 50 μmol/l for ifosfamide and 5 μmol/l for doxorubicin. The $IC_{50}$ values for samples of fibromatosis and soft tissue sarcoma were compared using the statistical computer software package SPSS (SPSS Inc., Chicago, IL) by the (non-parametric) Mann–Whitney $U$-test which uses variable rank and so is not affected by the distortion of the STS sample mean which was produced by limiting maximum $IC_{50}$ values.

### Results

Out of 97 fresh tissue samples collected, the diagnosis of fibromatosis was made in four cases. A photograph of fibromatosis cell growth in culture is shown in Fig. 1(a) and the corresponding histological section in Fig. 1(b). The characteristics of the patients are detailed in Table 1 and the corresponding chemosensitivity testing results are presented in Table 2. The mean $IC_{50}$ for doxorubicin in cases of fibromatosis was 0.35 μmol/l. The mean $IC_{50}$ for ifosfamide was 6.2 μmol/l. The corresponding values for STS samples were 1.69 and 14.8 μmol/l, respectively. Using the Mann–Whitney $U$-test there was a significant difference between the $IC_{50}$ values in STS and fibromatosis for ifosfamide ($p < 0.01$) but not for doxorubicin ($p = 0.1$). Comparative values for the two drugs are presented in Fig. 2(a) and (b).

**Table 2.** *Chemosensitivity testing results for fibromatosis patients*

| Study number | $IC_{50}$ doxorubicin (μmol/l) | $IC_{50}$ ifosfamide (μmol/l) |
|---|---|---|
| 3 | 0.36 | 5.8 |
| 41 | 0.65 | 12.9 |
| 44 | 0.27 | 2.1 |
| 91 | 0.13 | 4.1 |
| Mean | 0.35 | 6.23 |
| Range | 0.13–0.65 | 2.1–12.9 |

### Discussion

Compared to a panel of soft tissue sarcoma cells in culture, the $IC_{50}$ values for fibromatosis cells exposed to ifosfamide and doxorubicin were lower and, for ifosfamide, this difference was statistically significant. In STS we have previously shown that a high value $IC_{50}$ for the drugs is associated with clinical drug resistance.[25,26] We have been unable to make a similar comparison for cases of fibromatosis, no patient has required systemic therapy. Previous reports have demonstrated the clinical activity of chemotherapy in advanced cases of fibromatosis. A widely used chemotherapy strategy is the 'Philadelphia' regimen of low-dose weekly methotrexate (50 mg/week) and vinblastine (10 mg/week).[20,22] The activity of more aggressive regimens based on doxorubicin and dacarbazine has also been demonstrated[21,23,27] and additionally, in children, the combination of dacarbazine with vincristine and cyclophosphamide (VAC) has been shown to be effective.[28] We did not have sufficient clinical material available to test fibromatosis cells against these other cytotoxic drugs.

**Table 1.** *Fibromatosis patient characteristics*

| | | | | |
|---|---|---|---|---|
| Study number | 3 | 41 | 44 | 91 |
| Age at diagnosis | 24 | 45 | 28 | 31 |
| Sex | Female | Male | Male | Female |
| Clinical setting of sample | First relapse (after 45 months) | Primary surgery | First relapse (after 23 months) | Primary surgery |
| Site of tumour | Intra-abdominal | Upper arm | Thigh | Abdominal wall |
| Tumour size (pathological) | 11.5 × 8 × 7.5 cm | 5 × 3 cm | 17 × 7 × 6 cm | 13 × 11.5 × 10.5 cm |
| Adjuvant treatment given? | No | No | Radiotherapy | No |
| Current status | Local relapse after 6 months. No further treatment given. Remains well with disease | Alive and disease free | Alive and disease free | Alive and disease free |
| Length of follow up | 21 months | 18 months | 18 months | 6 months |

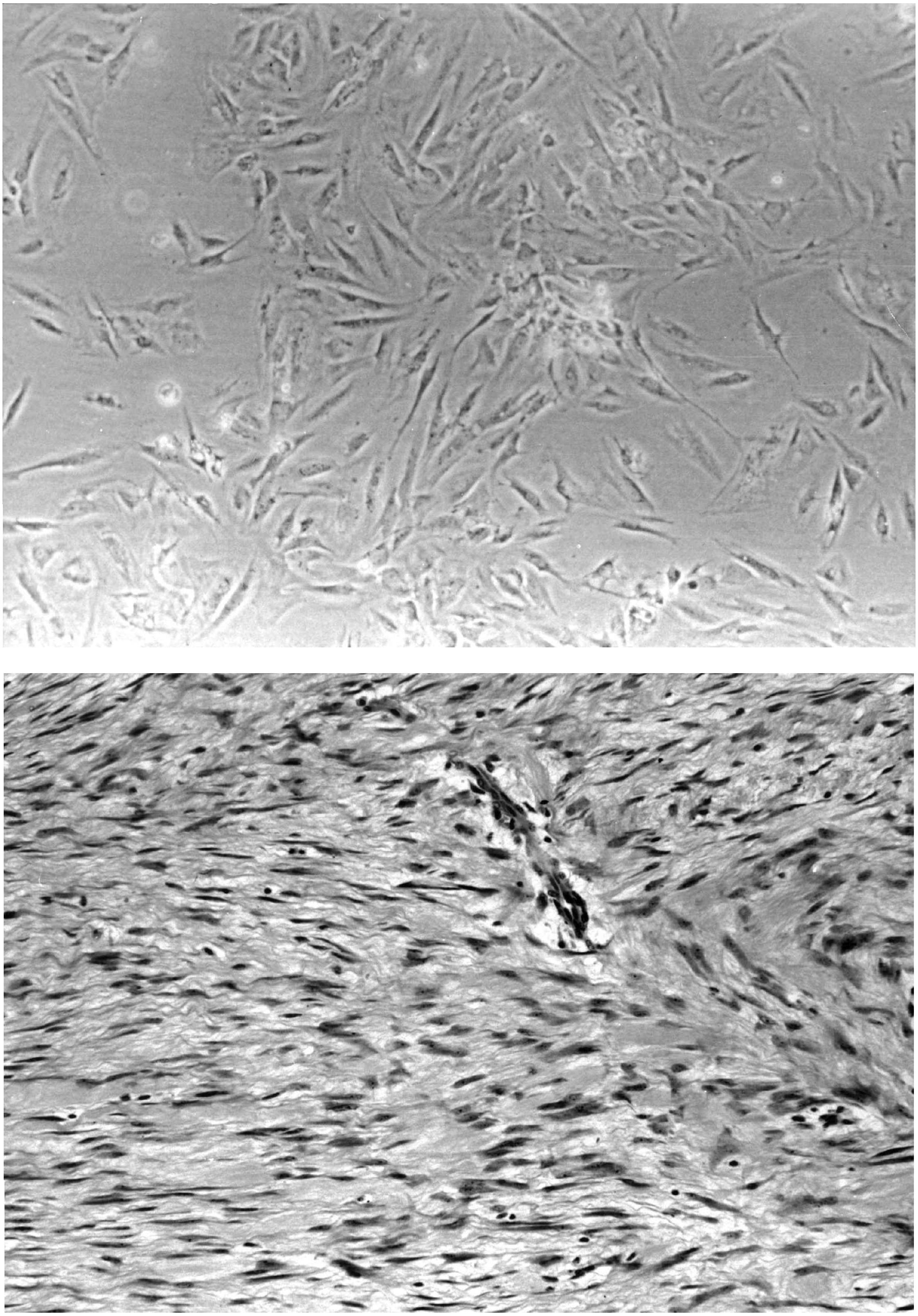

**Fig. 1.** *(a) Fibromatosis cells growing in tissue culture flask (unstained). (b) Histological section of fibromatosis sample from which cells in Fig. 1(a) were derived (H&E).*

Our *in vitro* observations support the inclusion of doxorubicin in regimens for the treatment of fibromatosis. We have also demonstrated the sensitivity of fibromatosis cells, at least in tissue culture, to ifosfamide. We are not aware of any other reports suggesting that ifosfamide is a useful drug in this condition, and therefore this introduces the possibility of a new approach to treatment using ifosfamide in first line drug combinations. For small, well-circumscribed lesions of fibromatosis, the treatment of choice should be surgery. Chemotherapy and radiation therapy should be avoided because of treatment-related

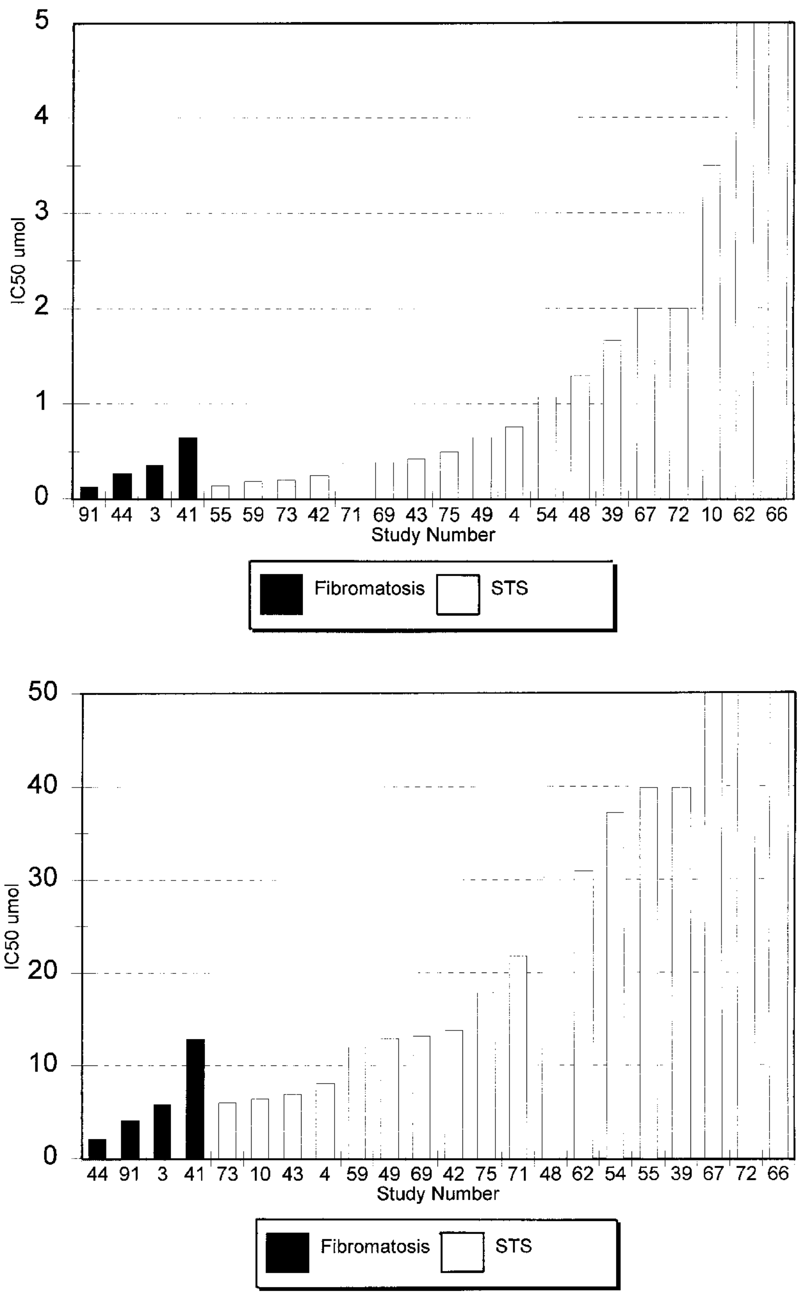

**Fig. 2.** *(a) $IC_{50}$ values after 72-h exposure to ifosfamide in fibromatosis and STS cells in culture using LDH assay. (b) $IC_{50}$ values after 72-h exposure to doxorubicin in fibromatosis and STS cells in culture using LDH assay.*

toxicity. However, for patients with inoperable progressive lesions, causing significant symptoms or posing a threat to normal organ function, it may be valuable to include ifosfamide in experimental treatment regimens.

### References

1 Enzinger FM, Weiss SW. *Soft tissue tumours*. St Louis: Mosby-Year Book Inc., 1995, pp. 201–229.
2 Hunt RTN, Morgan HC, Ackerman LV. Principles in the management of extraabdominal desmoids. *Cancer* 1960; 13:825.
3 McKinnon JG, Neifeld JP, Kay S, *et al.* Management of desmoid tumours. *Surg Gynecol Obstet* 1989; 169:104.
4 Posner MC, Shiu M, Newsome JL, *et al.* The desmoid tumour: not a benign disease. *Arch Surg* 1989; 124:191.
5 Enzinger FM, Shiraki M. Musculo-aponeurotic fibromatosis of the shoulder girdle (extra-abdominal desmoid): analysis of 30 cases followed up for 10 or more years. *Cancer* 1967; 21:1131.
6 Kofoed H, Kamby C, Agnostaki L. Aggressive fibromatosis. *Surg Gynecol Obstet* 1985; 160:124.
7 Brodsky JT, Gordon MS, Hadju SI, *et al.* Desmoid tumours of the chest wall: a locally recurrent problem. *J Thorac Cardiovasc Surg* 1992; 104:900.
8 Easter DW, Halasz NA. Recent trends in the management of desmoid tumours: Summary of 19 cases and review of the literature. *Ann Surg* 1989; 210:765.
9 Markhede G, Lundgren L, Bjurstam N, *et al.* Extraabdominal desmoid tumours. *Acta Orthop Scand* 1986; 57:1.
10 Rock MG, Pritchard DJ, Reiman HM, *et al.* Extraabdominal desmoid tumours. *J Bone Jt Surg* 1984; 66A:1369.

11 Sherman NE, Romsdahl M, Evans H, *et al.* Desmoid tumours: a 20 year radiotherapy experience. *Int J Radiat Oncol Biol Phys* 1990; 19:37.
12 McCullough WM, Parsons JT, van der Griend R, *et al.* Radiation therapy for aggressive fibromatosis: the experience at the University of Florida. *J Bone Jt Surg* 1991; 73(A):717.
13 Kiel KD, Suit HD. Radiation therapy in the treatment of aggressive fibromatosis (desmoid tumour). *Cancer* 1984; 54:2051.
14 Liebel SA, Wara WM, Hill DR, *et al.* Desmoid tumours: local control and patterns of relapse following radiation therapy. *Int J Radiat Oncol Biol Phys* 1983; 9:1167.
15 Kinzbrunner B, Ritter S, Domingo J, *et al.* Remission of rapidly growing desmoid tumour after tamoxifen therapy. *Cancer* 1983; 52:2201.
16 Waddell WR, Gerner RE, Reich MP. Non-steroidal anti-inflammatory drugs and tamoxifen for desmoid tumours and carcinomas of the stomach. *J Surg Oncol* 1983; 22:197.
17 Wilcken N, Tattersall MH. Endocrine therapy for advanced desmoid tumours. *Cancer* 1991; 68:1384.
18 Bauernhofer T, Stoger H, Schmid M, *et al.* Sequential treatment of recurrent mesenteric desmoid tumor. *Cancer* 1996; 77:1061–5.
19 Lanari A. Effect of progesterone on desmoid tumours (aggressive fibromatosis). *New Engl J Med* 1983; 309:1523.
20 Weiss AJ, Lackman RD. Low dose chemotherapy of desmoid tumours. *Cancer* 1989; 64:1192–4.
21 Patel SR, Evans HL, Benjamin RS. Combination chemotherapy in adult desmoid tumors. *Cancer* 1993; 72:3244–7.
22 Weiss AJ, Lackman RD. Therapy of desmoid tumors, fibromatosis, and related neoplasms. *Int J Oncol* 1995; 7:773–6.
23 Hamilton L, Blackstein M, Berk T, *et al.* Chemotherapy for desmoid tumours in association with familial adenomatous polyposis: a report of three cases. *Can J Surg* 1996; 39:247–52.
24 Coley HM, Lewandowicz G, Sargent J, Verrill MW. Chemosensitivity of Fresh and Continuous Tumour Cell Lines Using Cellular Lactate Dehydrogenase. *Anticancer Res* 1997; 17:231–6.
25 Coley HM, Verrill MW, Gregson SE, Judson IR. Drug resistance mechanisms in adult soft tissue sarcomas (STS) using primary and established cultures. *Proc Annu Meet Am Assoc Cancer Res* 1996; 37:311–2.
26 Coley HM, Verrill MW, Judson IR, Fisher C. p53 abnormalities influence modulation of Multi Drug Resistance (MDR) in primary cultures of adult soft tissue sarcoma. *Proc Annu Meet Am Assoc Cancer Res* 1997; 38:389–90.
27 Lynch HT, Fitzgibbons RJ, Chong S, *et al.* Use of doxorubicin and dacarbazine for the management of unresectable intra-abdominal desmoid tumors in gardner's syndrome. *Dis Colon Rectum* 1994; 37:260–7.
28 Raney B, Evans A, Granowetter R, Schnaufer L, Uri A, Littman P. Nonsurgical management of children with recurrent or unresectable fibromatosis. *Paediatrics* 1987; 79:394–8.
